# Acute Kidney Injury Outcomes of Elderly and Nonelderly Patients in the Medical Intensive Care Unit of a University Hospital in a Developing Country

**DOI:** 10.1155/2020/2391683

**Published:** 2020-01-30

**Authors:** Wanjak Pongsittisak, Kashane Phonsawang, Solos Jaturapisanukul, Surazee Prommool, Sathit Kurathong

**Affiliations:** ^1^Renal Division, Department of Internal Medicine, Faculty of Medicine Vajira Hospital, Navamindradhiraj University, Bangkok, Thailand; ^2^Renal Unit, Department of Internal Medicine, Sirindhorn Hospital, Bangkok, Thailand

## Abstract

**Background:**

Aging is associated with a high risk of acute kidney injury (AKI), and the elderly with AKI show a higher mortality rate than those without AKI. In this study, we compared AKI outcomes between elderly and nonelderly patients in a university hospital in a developing country.

**Materials and Methods:**

This retrospective cohort study included patients with AKI who were admitted to the medical intensive care unit (ICU) between January 1, 2012, and December 31, 2017. The patients were divided into the elderly (eAKI; age ≥65 years; *n* = 158) and nonelderly (nAKI; *n* = 142) groups. Baseline characteristics, comorbidities, principle diagnosis, renal replacement therapy (RRT) requirement, hospital course, and in-hospital mortality were recorded. The primary outcome was in-hospital mortality.

**Results:**

The eAKI group included more females, patients with higher Acute Physiology and Chronic Health Evaluation II scores, and patients with more comorbidities than the nAKI group. The etiology and staging of AKI were similar between the two groups. There were no significant differences in in-hospital mortality (*p*=0.338) and RRT requirement (*p*=0.802) between the two groups. After adjusting for covariates, the 28-day mortality rate was similar between the two groups (*p*=0.654), but the 28-day RRT requirement was higher in the eAKI group than in the nAKI group (*p*=0.042).

**Conclusion:**

Elderly and nonelderly ICU patients showed similar survival outcomes of AKI, although the elderly were at a higher risk of requiring RRT.

## 1. Introduction

The elderly population is increasing worldwide. Elderly patients often present with multiple comorbidities, such as diabetes mellitus, hypertension, chronic kidney disease (CKD), and coronary artery disease. Moreover, aging can negatively affect the ability to protect against cellular injuries and impair repair processes, resulting in poor outcomes among elderly patients.

Acute kidney injury (AKI)—a common disease burden worldwide—affects morbidity and mortality [1, 2]. Elderly individuals are susceptible to AKI, particularly when critically ill [3, 4]. This can be attributed to comorbidities, increased severity of acute illnesses, polypharmacy, need for invasive procedures, and age-dependent changes in this population [5–7]. Majority of the studies including elderly patients have revealed that the elderly with AKI show higher mortality than those without AKI [8–10]. Similar findings have also been reported in other age groups [11].

In case of the scarcity of resources, such as in developing countries, critically ill elderly patients with AKI may be treated with less intensive care to spare resources for young patients. Therefore, the present study aimed to compare the in-hospital outcomes of elderly and nonelderly patients with AKI admitted to the medical intensive care unit (ICU) of a university hospital in a developing country.

## 2. Materials and Methods

### 2.1. Study Population and Data Collection

This was a single-center retrospective cohort study conducted at a university hospital located in the center of Thailand. All patients who were admitted to the medical ICU between January 1, 2012, and December 31, 2017, were included. The inclusion criteria were as follows: age ≥18 years; admission to the medical ICU; and diagnosis of AKI according to the serum creatinine (sCr) criteria defined by Kidney Disease: Improving Global Outcomes [12]. AKI diagnosis was reviewed and confirmed for all the enrolled patients. The exclusion criteria were as follows: end-stage renal disease with concurrent renal replacement therapy (RRT); palliative care; pregnancy; kidney transplantation; advanced stage of malignancy; and substantially incomplete data. The following data were extracted for each patient from electronic and paper medical records: age; sex; weight; height; comorbidities; principal diagnosis; baseline laboratory data (blood urea nitrogen, sCr, hematocrit, arterial blood gas, sodium, potassium, chloride, bicarbonate, albumin, and lactate); Acute Physiology and Chronic Health Evaluation II (APACHE II) score, AKI etiology; AKI severity; vasopressor requirement; diuretic drug use; and mechanical ventilation requirement. The patients were classified into two groups based on age: elderly (eAKI; age ≥65 years) and nonelderly (nAKI; age <65 years).

### 2.2. AKI Diagnosis

AKI was defined as a 1.5-fold increase in sCr from the baseline level within 7 days or sCr ≥ 0.3 mg/dL within 48 h. The baseline sCr level was established based on one of the following criteria, in that order: (1) the lowest sCr within 7 days prior to AKI diagnosis; (2) the mean sCr level during 8–365 days prior to AKI diagnosis; or (3) the baseline sCr derived using the Modification of Diet in Renal Disease Study equation with an estimated glomerular filtration rate (eGFR) of 75 mL/min/1.73 m^2^ in the absence of a previous report of sCr level.

### 2.3. Primary and Secondary Outcomes

The primary outcome was in-hospital mortality. The secondary outcomes were RRT requirement, 28-day in-hospital mortality, combined 28-day mortality and RRT requirement, ICU stay duration, and time to RRT.

### 2.4. Ethical Considerations

This study was approved by the institutional review board of Navamindradhiraj University (COA 44/62) and was registered in the Thailand Clinical Trial Registry (TCTR20190606012). The requirement of patient consent was waived due to the retrospective nature of the study.

### 2.5. Statistical Analysis

Continuous variables are presented as mean with standard deviation or median with interquartile range (IQR), depending on data distribution by Shapiro–Wilk test. Categorical variables are presented as numbers and percentages. For comparisons, independent *t*-test was used when the data were normally distributed and the Mann–Whitney *U* test when the data were non-normally distributed. Chi-squared or Fisher's exact test was used for comparing categorical variables. Overall survival was plotted using Kaplan–Meier curves, and the groups were compared using the log-rank test. The association of age with 28-day mortality or RRT requirement was evaluated using Cox proportional hazards models. Data from all the patients were censored at the time of death, at hospital discharge, or at day 28, whichever occurred first. A two-sided *p* < 0.05 was considered statistically significant. All statistical analyses were performed using R version 3.4.4 (R Foundation for Statistical Computing, Vienna, Austria).

## 3. Results

### 3.1. Patient Characteristics

**Figure 1:**
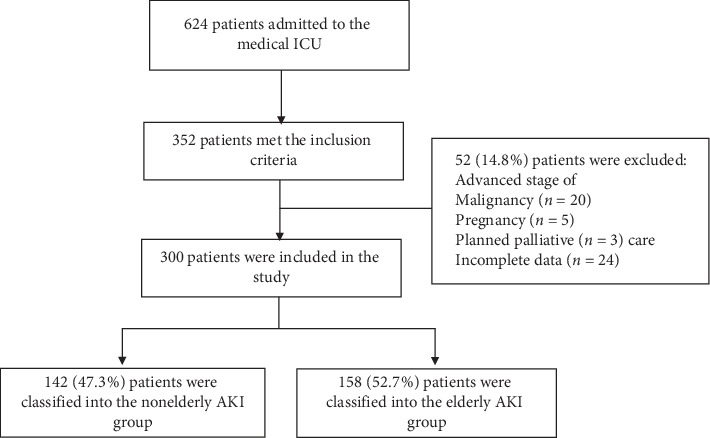
Screening and enrolment flow diagram.

During the 6-year study period, 624 patients were admitted to the medical ICU, of which 352 (56.4%) met the inclusion criteria. Of the 352, 52 were excluded (Figure 1). Finally, 300 patients with AKI were included in this study, of which 158 (47.3%) were classified in the eAKI and 142 (52.7%) in the nAKI group.  Table 1 summarizes the comparison of characteristics between the two groups. The eAKI group had a significantly higher proportion of females than the nAKI group and presented with more comorbidities (diabetes mellitus, hypertension, coronary artery disease, and CKD). The baseline renal function was lower, and CKD stage was more advanced in the eAKI group than in the nAKI group. There were 28 (eAKI, 12 (7.6%); nAKI, 16 (11.3%)) patients that did not have sCr result in 365 days prior to AKI diagnosis. The leading etiology of ICU admission was infection and/or sepsis, which was significantly more frequent in the eAKI group (62.6%) than in the nAKI group (48.6%). The etiology of AKI was similar in both groups. The patients in the eAKI group showed higher APACHE II scores and more frequent diuretic use. However, the staging and etiology of AKI were comparable between the two groups.

### 3.2. Mortality

**Figure 2:**
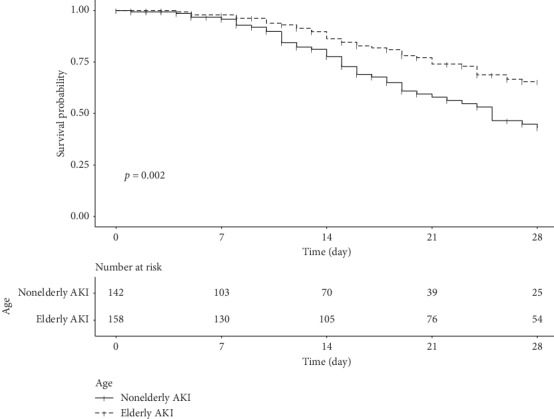
Kaplan–Meier survival curves of the elderly and nonelderly patients with AKI. There was a significant difference in survival between the groups (*p*=0.002, log-rank test).

Table 2 summarizes the outcomes of the patients in the two groups. Of the 300 patients with AKI, 113 (37.7%) suffered in-hospital mortality, of which 55 (34.8%) were in the eAKI group and 58 (40.9%) were in the nAKI group (*p*=0.34). The incidence of in-hospital mortality was 13.6, 10.6, and 18.6 deaths per patient-year in the overall cohort, eAKI group, and nAKI group, respectively. Median time to death was 19 (12–29) days. A total of 84 (28%) patients suffered 28-day in-hospital mortality. The Kaplan–Meier curves for 28-day in-hospital mortality are shown in Figure 2. The 28-day hospital mortality rate was significantly higher in the nAKI group than in the eAKI group (46 (32.4%) vs 38 (24.1%); log-rank *p*=0.002). The unadjusted Cox proportional hazard model showed that the 28-day in-hospital mortality rate was lower in the eAKI group than in the nAKI group (Table 3). However, there was no statistically significant difference in the 28-day in-hospital mortality rate between the groups after applying model II, which was adjusted for model I (adjusted with confounders: comorbidity, sex, weight, height, and baseline eGFR) plus the Charlson Comorbidity Index, presence of infection, APACHE II score, diuretics use, and AKI stage (hazard ratio (HR), 0.874; 95% confidence interval (CI), 0.484–1.577; *p*=0.654).

### 3.3. Secondary Outcomes

A similar proportion of patients in both groups required RRT (eAKI, 42 (26.6%); nAKI, 35 (24.6%); *p*=0.802), and the median time to RRT was also similar between the two groups. The indications for RRT are listed in Table 2. Five patients in eAKI and 4 patients in nAKI were performed RRT by continuous renal replacement therapy initially. The remaining were done conventional hemodialysis or sustained low-efficiency dialysis which depends on hemodynamic status. The patients in the eAKI group showed a significantly longer duration of ICU stay than those in the nAKI group (9 (4–17) vs 5 (2–10) days; *p*=0.0005). The rates of combined 28-day in-hospital mortality and RRT requirement were not significantly different between the two groups (*p*=0.766). The unadjusted Cox proportional hazard model showed similar rates of 28-day RRT requirement between the two groups. However, after applying model II, the eAKI group showed a significantly higher rate of 28-day RRT requirement than the nAKI group (HR, 1.989; 95% CI, 1.025–3.859; *p*=0.042).

## 4. Discussion

The present study compared the outcomes of elderly and nonelderly patients with AKI admitted to the medical ICU of a university hospital in a developing country. The results showed that the elderly showed more comorbidities and higher AKI severity than the nonelderly; however, after adjusting for covariates, there was no significant difference in the mortality rates of AKI between the two groups. Nevertheless, the elderly patients required prolonged ICU stay.

Furthermore, the incidence of AKI in our medical ICU was similar to that reported in previous studies [2, 11, 13]. Moreover, as reported previously [14–21], the incidence of AKI was higher in the elderly patients. Possible explanations for this include depleted nephron reserves, increased susceptibility to exposure to nephrotoxic agents, impaired ability to repair injuries, polypharmacy, and multiple comorbidities, specifically CKD [5, 22, 23]. Furthermore, elderly individuals are susceptible to severe infection and are therefore more likely to develop septic acute tubular necrosis (ATN) and experience mortality [3]. Indeed, infection and septic ATN were the leading etiologies of ICU admission and AKI in the present study, which is consistent with the trends in previous studies [8, 14, 24–26].

There is considerable evidence that irrespective of their age, patients with AKI show a higher mortality rate than those without AKI [8–10]. In a previous study, age was an independent predictor of mortality risk in AKI patients [11]. On the contrary, some studies have reported that age was not an independent risk factor for mortality in AKI patients >60 years of age [8, 27]. The mortality rate of elderly patients with AKI has been reported to be 18%–61% [8, 28–30]; this wide range could be due to the heterogeneity of the study populations, availability of a critical care unit, standard of care administered, or research methodology used.

The present study reported similar in-hospital mortality rates between the elderly and nonelderly patients with AKI. This might be explained by the pattern of clinical practice for selecting patients for admission to our medical ICU. Because of resource constraints, the patients are carefully selected according to a number of criteria, one of which is age. Typically, older patients are admitted to the ICU when their previous health seems to be favorable and their concurrent illness is not anticipated to leave them moribund. Conversely, younger patients are likely to be admitted to the ICU regardless of their poor previous health status or a moribund state. Another possible explanation is the difference between a patient's chronological and biological age; younger people with unfavorable lifestyle and poor medical history may be at an increased risk of mortality. Several studies showed frailty in elderly patients was associated with increased risk and poor outcome of AKI [31–34]. By the concept of frailty, this reflects biological age rather than chronological age. Hence, there is an increasing number of studies to evaluate frailty in nonelderly patients [35]. Unfortunately, the present study did not have clinical frailty score data to explore this question.

In this study, after adjusting for covariates, the elderly patients were more likely to require RRT than the nonelderly patients. As mentioned earlier, this might be explained by the depleted nephron reserves and impaired ability to repair injuries in the elderly.

The present study has some limitations. This was a retrospective study, which may have led to the omission of some clinical information. Moreover, the enrollment was limited to patients admitted to our medical ICU. Finally, this was a single-center study including a relatively small cohort. Nonetheless, the present study represented outcomes in the real-world clinical settings in a developing country.

In conclusion, in a resource-limited setting, as seen in developing countries, the survival outcomes of elderly patients with AKI are no worse than those of younger patients with AKI, although the elderly require prolonged ICU stays. Therefore, in elderly patients with AKI, careful patient selection for ICU admission and intensive care are imperative to achieve excellent outcomes. Further large-scale prospective studies may be required to confirm our findings.

## Figures and Tables

**Table 1:** Baseline characteristics and clinical and laboratory parameters of the patients with AKI^†^.

Parameter	Overall	Nonelderly	Elderly	*p*
Number	300	142	158	
Age, year	66 (52–77)	51 (42–59)	76 (70–82)	<0.0001
Sex, male, *n* (%)	166 (55.3)	91 (64.1)	75 (47.5)	0.0055
Weight (kg)	60 (50–70)	62 (55–70)	59 (50–65)	0.0012
Height (cm)	160 (155–168)	165 (158–170)	160 (154–165)	0.0001
BMI (kg/m^2^)	22.9 (20.2–25.8)	23.6 (20.0–27.3)	22.2 (20.4–25.4)	0.19
Comorbidity, *n* (%)				
DM	126 (42.0)	47 (33.1)	79 (50.0)	0.0045
HTn	181 (60.3)	55 (38.7)	126 (79.8)	<0.0001
CAD	43 (14.3)	8 (5.6)	35 (22.2)	<0.0001
CKD	198 (66)	70 (49.4)	128 (81)	<0.0001
CKD stage, *n* (%)				<0.0001
I-II	75 (37.9)	30 (42.9)	45 (35.2)	
IIIa	48 (24.2)	16 (22.9)	32 (25.0)	
IIIb	33 (16.7)	12 (17.1)	21 (16.4)	
IV	42 (21.2)	12 (17.1)	30 (23.4)	
eGFR, mL/min/1.73 m^2^	72.5 (45–95)	89.5 (56–104)	57.9 (38–82)	<0.0001
Baseline sCr, mg/dL	1.00 (0.79–1.30)	0.97 (0.76–1.13)	1.00 (0.85–1.46)	0.0018
Charlson Comorbidity Index, points	4 (2–6)	2 (1–4)	5 (4–7)	<0.0001
Principle diagnosis, *n* (%)				0.0196^‡^
Infection or sepsis	168 (56.0)	69 (48.6)	99 (62.6)	
ACS or HF	27 (9.0)	11 (7.8)	16 (10.1)	
Malignancy	25 (8.3)	16 (11.3)	9 (5.7)	
GI bleeding	22 (7.3)	16 (11.3)	6 (3.8)	
Hyperglycemic crisis	15 (5.0)	9 (6.3)	6 (3.8)	
Stroke	12 (4.0)	9 (6.3)	3 (1.9)	
Cirrhosis	5 (1.7)	3 (2.1)	2 (1.3)	
Other	26 (8.7)	9 (6.3)	17 (10.8)	
APACHE II score, points	26.0 (20–32)	24.0 (20–30)	27.5 (22–33)	0.001
Ventilator, *n* (%)	269 (89.7)	126 (88.8)	143 (90.5)	0.75
ARDS, *n* (%)	27 (9.0)	13 (9.2)	14 (8.8)	1.00
Need vasopressors, *n* (%)	202 (67.3)	95 (66.9)	107 (67.7)	0.97
Diuretic used, *n* (%)	172 (57.3)	65 (45.8)	107 (67.7)	0.0002
sCr at ICU, mg/dL	2.15 (1.60–3.11)	2.10 (1.52–3.06)	2.20 (1.65–3.18)	0.33
Hematocrit, %	29.9 (25.1–35.9)	29.9 (24.3–37.6)	29.8 (25.3–34.0)	0.77
Albumin, g/dL	2.7 (2.2–3.3)	2.8 (2.3–3.4)	2.6 (2.2–3.2)	0.15
Bicarbonate, mmol/L	17 (12–21)	16 (12–21)	17 (13–21)	0.17
Lactate, mmol/L	4.4 (2.6–7.6)	4.7 (3.2–8.9)	4.2 (2.2–7.2)	0.06
AKI stage, *n* (%)				0.59
I	83 (27.7)	36 (25.4)	47 (29.7)	
II	80 (27.7)	37 (26.0)	43 (27.2)	
III	137 (45.6)	69 (48.6)	68 (43.1)	
AKI etiology, *n* (%)				
Septic AKI	116 (38.7)	53 (37.3)	63 (39.9)	
ATN	87 (29.0)	41 (28.9)	46 (29.1)	
Hypovolemia	59 (19.7)	33 (23.2)	26 (16.5)	
CRS	20 (6.7)	6 (4.3)	14 (8.9)	
Nephrotoxic ATN	8 (2.7)	0	8 (5.1)	
Other	10 (3.2)	9 (6.3)	1 (0.5)	

Data are presented as median (interquartile range) or number (percentage). ^†^The elderly were those aged ≥65 years. ^‡^Chi-square test for infection or sepsis versus no infection or sepsis. ACS, acute coronary syndrome; AKI, acute kidney injury; APACHE II, Acute Physiology and Chronic Health Evaluation II; ARDS, acute respiratory distress syndrome; ATN, acute tubular necrosis; BMI, body mass index; CAD, coronary artery disease; CKD, chronic kidney disease; CRS, cardiorenal syndrome; DM, any type of diabetes mellitus; eGFR, estimated glomerular filtration rate; GI, gastrointestinal; HF, heart failure; HTn, hypertension; ICU, intensive care unit; and sCr, serum creatinine.

**Table 2:** Comparison of outcomes between the nonelderly and elderly patients with AKI^†^.

Outcomes	Overall	Nonelderly	Elderly	RR (95%CI)	*p*
In-hospital mortality, *n* (%)	113 (37.67)	58 (40.85)	55 (34.81)	0.88 (0.69–1.11)	0.34
28-day in-hospital mortality, *n* (%)	84 (28.0)	46 (32.4)	38 (24.1)	0.81 (0.64–1.04)	0.14
Requiring RRT, *n* (%)	77 (25.7)	35 (24.6)	42 (26.6)	1.06 (0.80–1.40)	0.80
28-day in-hospital mortality and RRT requirement, *n* (%)	172 (57.3)	69 (48.6)	73 (46.2)	0.95 (0.75–1.20)	0.77
Time to death, days	19 (12–29)	17 (11–25)	21 (14–33)		0.11
Length of ICU stay, days	6 (3–15)	5 (2–10)	9 (4–17)		0.0005
Time to initiated RRT, days	2 (1–3)	2 (1–3)	2 (1–4)		0.90
Indication for RRT, *n* (%)					0.30
Acidosis	29 (37.7)	17 (48.6)	12 (28.6)		
Fluid overload	26 (33.8)	8 (22.9)	18 (42.9)		
Uremia	16 (20.7)	8 (22.9)	8 (19.0)		
Electrolyte disturbances	6 (7.8)	2 (5.6)	4 (9.5)		

Data are presented as median (interquartile range) or number (percentage). ^†^The elderly were those aged ≥65 years; abbreviations: AKI, acute kidney injury; CI, confidence interval; ICU, intensive care unit; RR, relative risk; and RRT, renal replacement therapy.

**Table 3:** Cox proportional hazard ratios for the primary and secondary outcomes of the elderly and nonelderly patients with AKI.

Outcome	Crude	*p*	Model I^†^	*p*	Model II^‡^	*p*
	HR (95% CI)		HR (95% CI)		HR (95% CI)	
28-day in-hospital mortality	0.51 (0.33–0.79)	0.0024	0.49 (0.30–0.81)	0.0062	0.87 (0.48–1.58)	0.65
28-day RRT requirement	0.99 (0.64–1.57)	0.99	0.91 (0.55–1.50)	0.70	1.99 (1.02–3.86)	0.042

^†^Model I: adjusted for comorbidity (DM, HTn, CAD, or CKD); sex, weight, height, and baseline eGFR. ^‡^Model II: Model I plus infection or sepsis, APACHE II score, diuretic use, and AKI stage. APACHE II, Acute Physiology and Chronic Health Evaluation II; AKI, acute kidney injury; CAD, coronary artery disease; CI, confidence interval; CKD, chronic kidney disease; DM, any type of diabetes mellitus; HR, hazard ratio; HTn, hypertension; and RRT, renal replacement therapy.

## Data Availability

The data used to support the findings of this study are available from the corresponding author upon request.
